# Narrowband and flexible perfect absorber based on a thin-film nano-resonator incorporating a dielectric overlay

**DOI:** 10.1038/s41598-020-74893-1

**Published:** 2020-10-20

**Authors:** Chul-Soon Park, Sang-Shin Lee

**Affiliations:** 1 Nano Device Application Center, Kwangwoon University, 20 Kwangwoon-ro, Nowon-gu, Seoul, 01897 South Korea; 2 Department of Electronic Engineering, Kwangwoon University, 20 Kwangwoon-ro, Nowon-gu, Seoul, 01897 South Korea

**Keywords:** Engineering, Materials science, Optics and photonics, Physics

## Abstract

We developed a flexible perfect absorber based on a thin-film nano-resonator, which consists of metal–dielectric–metal integrated with a dielectric overlay. The proposed perfect absorber exhibits a high quality (Q-)factor of ~ 33 with a narrow bandwidth of ~ 20 nm in the visible band. The resonance condition hinging on the adoption of a dielectric overlay was comprehensively explored by referring to the absorption spectra as a function of the wavelength and thicknesses of the overlay and metal. The results verified that utilizing a thicker metal layer improved the Q-factor and surface smoothness, while the presence of the overlay allowed for a relaxed tolerance during practical fabrication, in favor of high fidelity with the design. The origin of the perfect absorption pertaining to zero reflection was elucidated by referring to the optical admittance. We also explored a suite of perfect absorbers with varying thicknesses. An angle insensitive performance, which is integral to such a flexible optical device, was experimentally identified. Consequently, the proposed thin-film absorber featured an enhanced Q-factor in conjunction with a wide angle of acceptance. It is anticipated that our absorber can facilitate seminal applications encompassing advanced sensors and absorption filtering devices geared for smart camouflage and stealth.

## Introduction

Thin-film nanostructures have been actively exploited for diverse functional devices. Their spectral transmission, reflection, and absorption can be aptly tailored owing to their outstanding merits, which include a simple structure, high efficiency, good scalability, a lithography-free fabrication process^1–6^, and easy fabrication on exotic flexible substrates^7–9^. As representative examples, a suite of color-filtering devices, drawing upon a thin-film nano-resonator, has been constructed to adaptively manipulate transmission and reflection in the visible band^9–15^.

Perfect absorbers, which absorb a specific spectral band and do not permit reflection or transmission, have recently gained considerable interest by virtue of their wide range of applications spanning photovoltaic cells^16^, photodetectors^17,18^, thermal imaging^19^, and sensors^20–28^. Perfect absorbers engaging various nanostructures (e.g., plasmonic and metasurface schemes)^18–27,29^ and multilayered structures^1–4,28,30–37^ are being extensively studied. Although narrowband absorbers are known to be suitable for sensing and absorption filtering^2,25–27^, conventional approaches have mostly focused on multiband^21,25^ or broadband absorption^31–37^ for realizing energy harvesting such as thermo-photovoltaics^2,17,34^. Narrowband absorbers, exhibiting a high quality factor (Q-factor) ranging from a few tens to hundreds, could be theoretically achieved by using nanostructured metamaterials even in the visible wavelength region^27,38,39^. Despite their narrow spectral bandwidth, plasmonic nanostructures have mostly been implemented to operate in the infrared region^20–23^. The major drawback of such nano-patterned structures is the complicated fabrication process. This includes sophisticated electron beam lithography, which is disadvantageous from the standpoint of large-scale applications such as flexible devices. Thus, lithography-free multilayered structures have recently attracted considerable interests because of their outstanding scalability^2–5,31–33^. Some schemes have been geared towards incorporating an absorbing medium like silicon (Si) and germanium (Ge)^40–46^. These highly absorptive materials are effective at suppressing a certain band in the visible spectrum to render vivid reflection colors^42–46^; however, there is a trade-off with the Q-factor associated with the nano-resonator. Approaches such as those that use photonic crystal, which are constructed by vertically stacking alternate layers of high- and low-index materials, can be adopted to improve the Q-factor^47–49^, while the thickness of each layer should be strictly controlled. Meanwhile, multilayered nanostructures made of both metal and dielectric have been perceived as promising candidates for spectral engineering required to boost the absorption and Q-factor. In particular, a Fabry–Pérot nano-resonator comprising a metal–dielectric–metal (MDM) configuration can act as an elegant perfect absorber in the presence of a lossless dielectric medium^1,12,50^. Nevertheless, MDM absorbers that are comprised of lossy metals including chromium (Cr), titanium (Ti), nickel (Ni), tungsten (W), and manganese (Mn), may feature a broadband absorption leading to a low Q-factor^2–4,32–34^. A viable approach to achieve higher Q-factor is using noble metals such as gold (Au) or silver (Ag)^1,12,30,50^. Metal such as Ag was chiefly utilized to realize high reflectivity and low extinction across the whole visible band^9–14,44^. However, the top metal surface is inevitably vulnerable to oxidation; this broadens the bandwidth, which reduces the Q-factor and degrades the performance. Moreover, with regard to the design of the nano-resonant structure, a constant metal thickness hampers control of the bandwidth.

In this article, we propose and demonstrate a narrowband perfect absorber based on a thin-film nano-resonator, which comprises an MDM structure tethered to a dielectric overlay, resembling a (metal–dielectric–metal)|dielectric (MDMD) configuration. A flexible perfect absorber resting on a pliable substrate has been also realized. The dielectric overlay helps mitigate the drawbacks of the MDM configuration (e.g., a fixed metal thickness and exposure of the surface to the environment) for bandwidth control and actual realization. The influences of both the metal thickness and dielectric overlay on the optical characteristics were meticulously addressed. The proposed device features an enhanced Q-factor in conjunction with a wide angle of acceptance, which are the primary concerns for a flexible perfect absorber. The proposed device is projected to be readily applicable to biosensors, smart camouflage, stealth, etc.

## Results and discussion

### Flexible perfect absorber based on MDMD configuration

**Figure 1:**
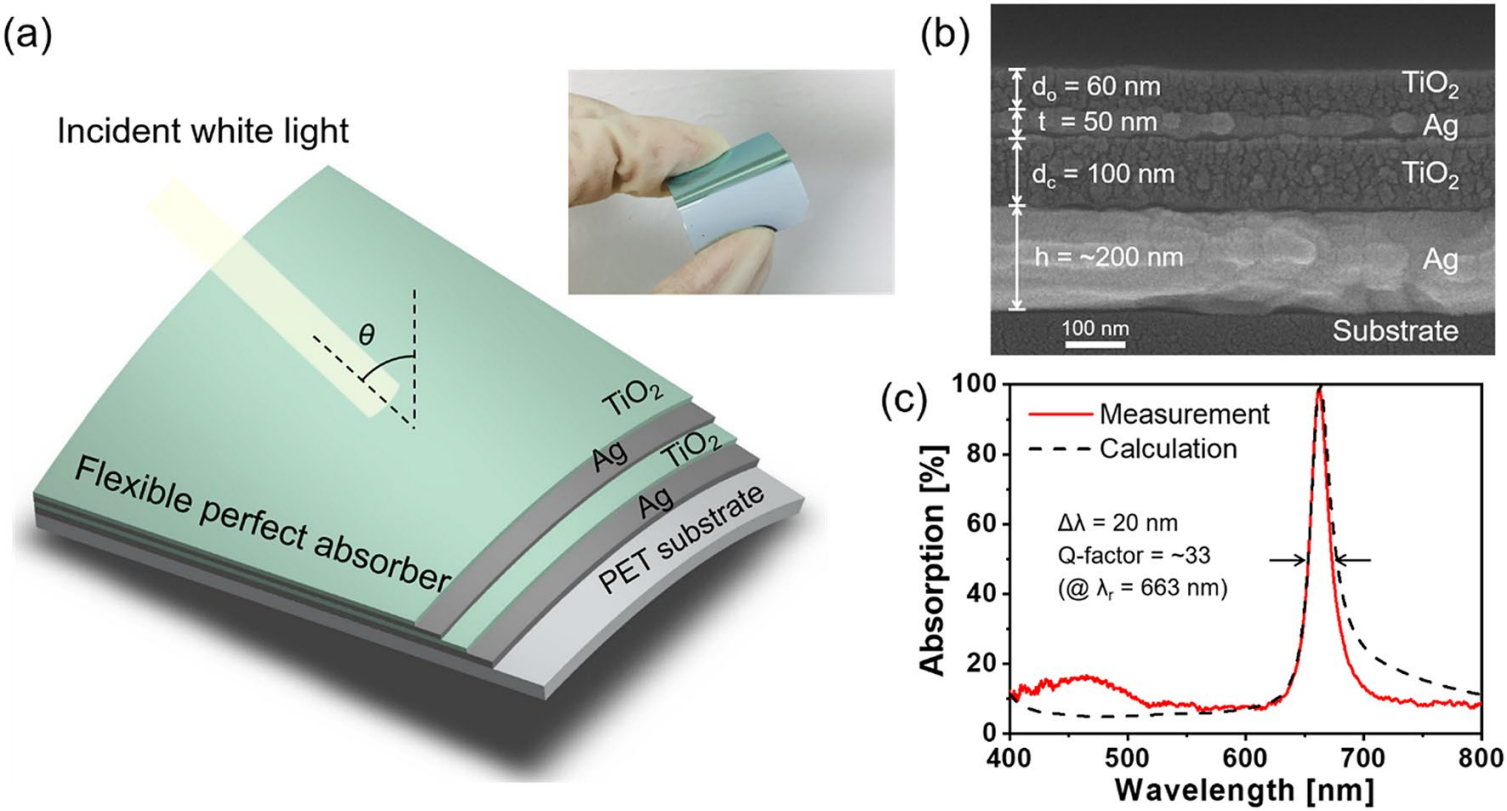
(**a**) Schematic of the proposed flexible perfect absorber; the inset shows the fabricated device. (**b**) Cross-sectional SEM image of the prepared absorber. (**c**) Measured and calculated absorption spectra in the visible band.

Figure 1a shows a schematic for the proposed flexible perfect absorber, where an Ag–TiO_2_–Ag nano-resonator is integrated with a titania (TiO_2_) dielectric overlay for the MDMD configuration^13,14^. The inset shows a sample of the perfect absorber fabricated on a flexible plastic substrate made of polyethylene terephthalate (PET). A cross-section of the device was observed under a high-resolution scanning electron microscope (Hitachi SEM S-4800), as depicted in Fig. 1b. The thickness of each layer can be identified in the captured image. The bottom Ag layer was set to a thickness (h) of ~ 200 nm so that incident light could be sufficiently reflected throughout the visible band and transmission through the device is prevented. The TiO_2_ cavity, top Ag layer, and TiO_2_ overlay were set to thicknesses of d_c_ = 100 nm, t = 50 nm, and d_o_ = 60 nm, respectively. Hence, perfect absorption could be achieved at a wavelength of λ = 663 nm. For the device fabrication, prior to the formation of thin films, organic and inorganic contaminants on a flexible PET substrate were removed through a series of ultrasonic treatment in acetone, ethanol, and deionized water. Ag and TiO_2_ films were successively deposited on the substrate via electron beam evaporation to cover a footprint of 25 × 25 mm^2^. The deposition rates for the Ag and TiO_2_ films were 0.3 and 0.1 nm/s, respectively.

The proposed perfect absorber was designed and assessed with a commercially available simulation tool: Essential Macleod (Version 9.8.437). The refractive indices of the materials used for the calculation are plotted in Fig. S1 of the Supplementary Information; these were extracted from separately grown films with the help of a spectroscopic ellipsometer (M-2000D, J. A. Woollam). Figure 1c shows the measured and calculated absorption spectra of the perfect absorber in the range of 400–800 nm. Complete absorption occurred at a resonant wavelength of λ_r_ = 663 nm in a narrow bandwidth of ∆λ = 20 nm, which indicates a high Q-factor of (λ_r_/∆λ) =  ~ 33. The measurement and calculation results showed good correlation. A small discrepancy in the bandwidth may be attributed to the fact that the Ag film was practically deposited to assume a slightly reduced refractive index compared with the simulation cases. The reflection spectrum was primarily investigated by using a spectrometer equipped with a reflection probe, which was devised to accommodate an optical beam normally reflecting off the samples. The absorption was then obtained according to the relationship A = 100 − R (%), where the transmission was assumed to be negligible for a thick bottom mirror made of Ag.

**Figure 2:**
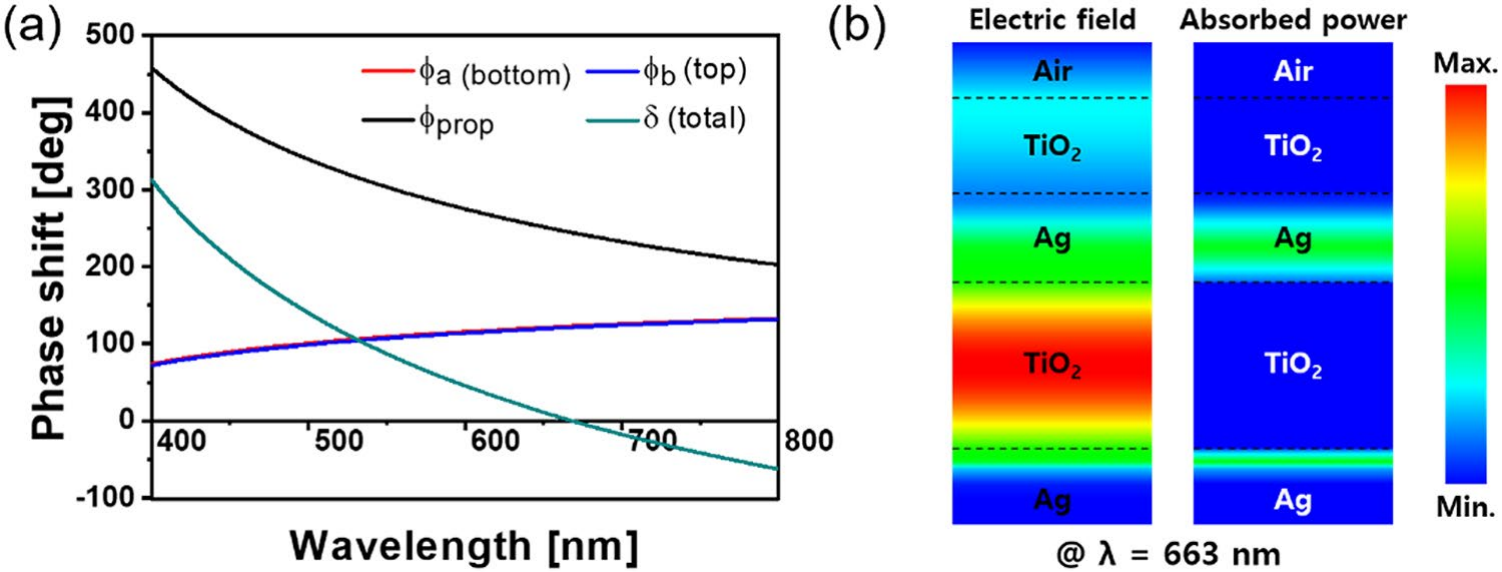
(**a**) Calculated phase shifts for thin-film nano-resonator. (**b**) Electric field and absorbed power profiles for thin-film perfect absorber at the resonant wavelength of λ_r_ = 663 nm.

For our absorber device, its complete absorption can be comprehended from the perspective of a Fabry–Pérot resonator^51^, whose resonance characteristics are elucidated in Fig. 2. The MDM configuration of the resonator should allow for strong resonance pertaining to the dielectric cavity. The reflection of concern can be expressed as $R = \frac{{2\sqrt {R_{a} R_{b} } (1 - \cos \delta )}}{{1 + R_{a} R_{b} - 2\sqrt {R_{a} R_{b} } \cos \delta }}$^13,14,51^. Here, *R*_*a*_ and *R*_*b*_ represent the reflectances corresponding to the bottom and top Ag–TiO_2_ interfaces, respectively. The total phase shift accumulated during a single roundtrip within the cavity is equivalent to $\delta = \phi_{prop} - (\phi_{a} + \phi_{b} )$, where $\phi_{a}$ and $\phi_{b}$ are the reflection phase shifts at the metal–dielectric interfaces. The roundtrip propagation phase shift is given by $\phi_{prop} = (4\pi /\lambda )nd_{c}$ for a normal incidence, where *n* and *d*_*c*_ represent the refractive index and cavity thickness, respectively. A minimum reflection is anticipated to arise at a resonant wavelength, where the condition $\delta = 2m\pi$ (*m* an integer) is satisfied. The absorption is derived from A = 1 − R − T (T = 0); thus, the optimum absorbance is achieved at the resonant wavelength, which indicates that a zero reflection is tantamount to perfect absorption. The resonance condition governed by the individual phase shifts pertaining to the nano-resonator is delineated Fig. 2a. Under the assumption that the top Ag layer is sufficiently thick and the TiO_2_ overlay contributes no considerable reflection phase shift, the reflection at the top Ag–TiO_2_ interface imposes the phase shift $\phi_{b}$, which is comparable to $\phi_{a}$. The propagation phase was calculated while the imaginary part of the refractive index was neglected. As expected, the total phase shift came close to zero at the resonance wavelength of 663 nm. Figure 2b shows the electric (E-)field and absorbed power profiles for the nano-resonator. For the MDM scheme, a strong Fabry–Pérot resonance was implied by the reinforced E-field profile in the dielectric cavity^1^. The optical power was mostly absorbed in the metallic layers rather the dielectric ones, considering that the optical loss is predominantly governed by the substantially higher extinction nature of the former compared with that of the latter^1,40,42^.

### Analysis of a narrowband perfect absorption enabled by a dielectric overlay

**Figure 3:**
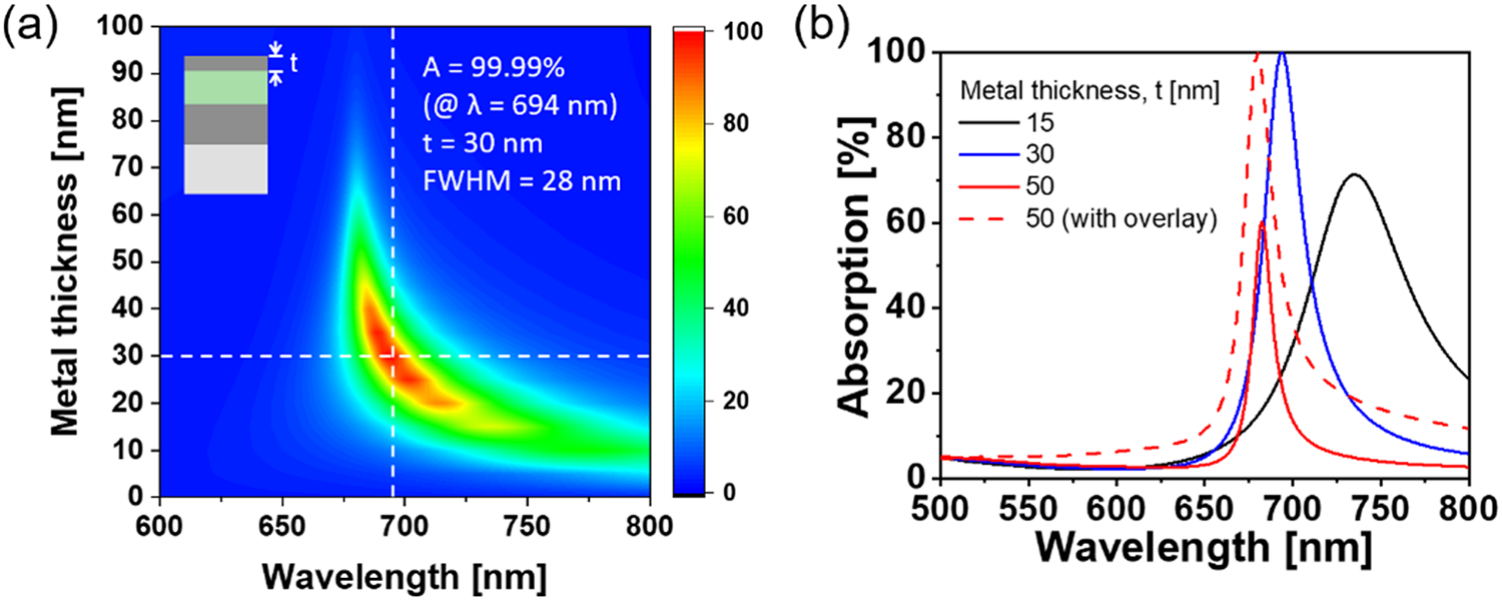
Absorption spectra according to different metal thicknesses. (**a**) Calculated absorption spectra for the MDM configuration as a function of wavelength and top Ag thickness (t) when d_c_ = 100 nm and h = 200 nm. (**b**) Expected spectra with different top Ag thicknesses t = 15, 30, and 50 nm. The absorption was enhanced when an overlay was adopted for t = 50 nm.

The resonance in conjunction with zero reflection, which is the condition for perfect absorption, was examined in detail at different thicknesses of the metallic layers with and without a dielectric overlay. The impact of the metal thickness was studied by tracking the absorption efficiency, as shown in Fig. 3. An Ag mirror was deemed to act as a good reflector across the visible band. When an additional dielectric layer was introduced to the Ag film, the thin-film structure formed an asymmetric Fabry–Pérot resonator in which a non-trivial phase shift was invoked by the metal substrate^40^. As can be inferred from Fig. S2 of the Supplementary Information, the total absorption associated with a lossless dielectric medium on metal is relatively small, compared with an absorbing medium such as Ge atop a metallic layer^40,42^. This is because the air–dielectric boundary renders no profound reflection, as in the case of the lossless dielectric. Consequently, placing an extra metal layer atop the dielectric was found to help invoke a strong resonance, which forged an MDM structure like the Fabry–Pérot resonator^51^. Figure 3a shows the calculated absorption spectra with respect to the metal thickness t for d_c_ = 100 nm and h = 200 nm. As addressed in previous approaches^1,30^, a strong resonance combined with enhanced absorption was secured for t =  ~ 30 nm with the MDM configuration involving an Ag mirror. Significant absorption beyond 99.9% was theoretically attained at λ = 694 nm with a full width at half maximum (FWHM) of 28 nm. This evidence shows that the resonance condition is critically sensitive to the metal thickness, which can shift the center wavelength and degrade the absorption efficiency.

Similarly, we manufactured another perfect absorber based on the MDM configuration and summarize its characterization results in Fig. S3a of the Supplementary Information, where the inset displays the prepared device on a PET substrate. The spectral bandwidth was slightly broader than expected, which can be attributed to scattering loss due to an unexpectedly rough Ag surface, as displayed in Fig. S3b. The Ag film made of nanoscale grains, which are commonly observed in an ultra-thin Ag film, is known to lead to a broader absorption band^31^. The uneven surface was mostly likely to reduce the Q-factor of the resonators and amplify the discrepancies in the designed and fabricated film thicknesses. For the evaporation process of Ag, many small isolated metallic particles formed atop the substrate and started clustering at a certain point. The voids between islands were deemed to be more faithfully filled with an increasing amount of deposited metal, thereby improving the uniformity of the reflective surface^52^. The roughness issue of ultra-thin Ag surface can be mitigated by using a wetting layer such as Ge or polymer^52–54^. However, an absorptive Ge layer may engender an undesired absorption. Otherwise, an additional process is required to coat the polymer.

We attempted to boost the Q-factor of the nano-resonator and mitigate the impact of the thickness variation and surface roughness of the Ag film. Thickening the metal film shrinks the bandwidth as a result of improved reflection at the metal–dielectric interface^14,51^. During the formation of Ag layers, the surface quality can improve in terms of flatness and smoothness by increasing the thickness of the metal, thus reducing the scattering loss. A dielectric overlay was introduced to serve as an anti-reflection (AR) coating. The dielectric overlay can also help prevent metal oxidation and promote absorption, as elaborated in Fig. 3b. When the thickness of the top Ag layer exceeds t = 30 nm, the bandwidth decreases with the absorption efficiency of the MDM nano-resonator. For an Ag thickness of t = 50 nm, the absorption spectra gave rise to a narrow bandwidth in tandem with decreased efficiency. Fortunately, when a dielectric overlay is atop the MDM nano-resonator (i.e., the MDMD configuration) increasing the metal thickness can improve the absorption efficiency up to ~ 100%.

**Figure 4:**
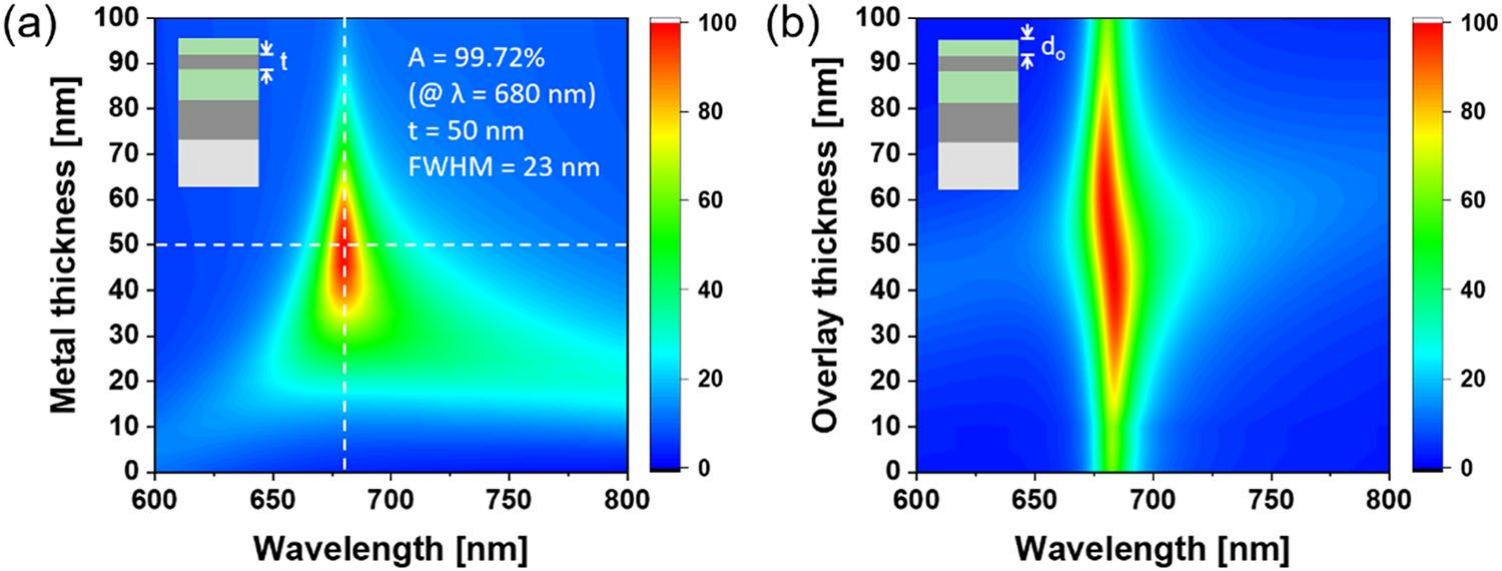
Calculated absorption spectra for the MDMD configuration according to (**a**) top Ag thickness with d_o_ = 60 nm and (**b**) overlay thickness with t = 50 nm; d_c_ and h were fixed at 100 nm and 200 nm, respectively.

To validate the effectiveness of the dielectric overlay, the absorption efficiency was examined with respect to the metal thickness (t) and overlay thickness (d_o_). Figure 4a shows the calculated absorption spectra with the metal thickness (t) under a TiO_2_ overlay of d_o_ = 60 nm. The determination of the overlay thickness at d_o_ = 60 nm is discussed later alongside the operation of the AR coating. The highest absorption of 99.7% at a wavelength of 680 nm was obtained for t =  ~ 50 nm, which is on a par with that of the MDM scheme. The effect of the overlay thickness was also scrutinized at t = 50 nm. The absorption was observed to improve with increasing overlay thickness, as shown in Fig. 4b. The overlay, which was optimized to a thickness of d_o_ =  ~ 60 nm, facilitated an absorption above 90% accompanied by a slight shift in the center wavelength in the range of d_o_ = 30–70 nm. The resonance wavelength was preserved to some extent against variations in the thicknesses of the metal and dielectric overlay. Considering the center wavelength is not drastically dependent on the thickness variations, an affordable fabrication tolerance can be secured for film deposition.

**Figure 5:**
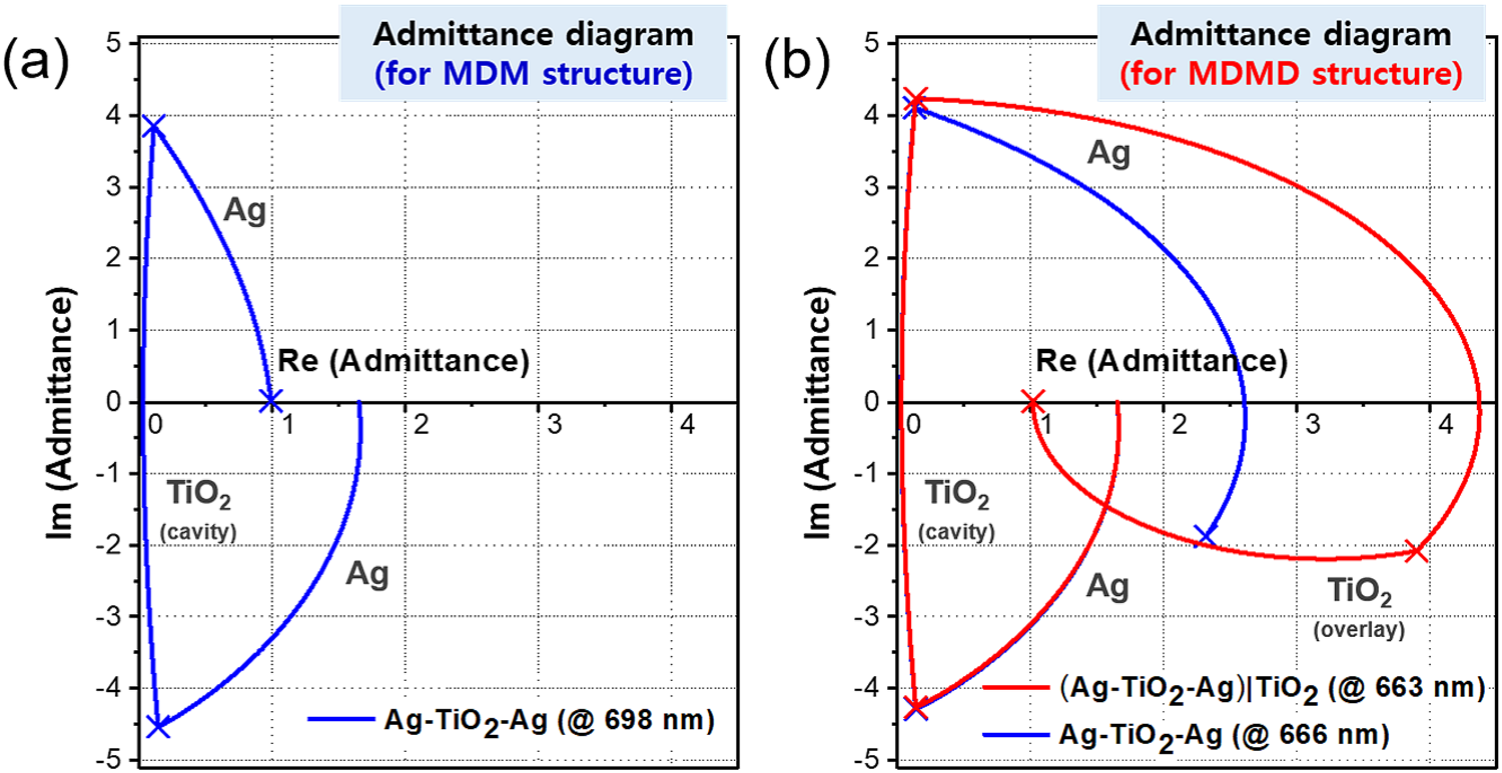
Calculated admittance diagrams for (**a**) MDM and (**b**) MDMD structures at resonance wavelengths.

The role played by a dielectric overlay acting as an AR coating can be efficiently interpreted by referring to an optical admittance diagram. The calculated admittances for the MDM and MDMD structures were traced at their resonance wavelengths, as shown in Fig. 5. The effective admittance in relation to the entire structure, as seen from the incident medium, is denoted as $Y = x + iy$, where x and y are the real and imaginary parts, respectively, of the admittance^55^. The reflection is determined by $R = r^{2} = \left( {\frac{1 - Y}{{1 + Y}}} \right)^{2}$ for an incident medium of air^55^. To reduce the reflection and thus maximize the absorption, the discrepancy needs to be minimized between the effective admittance of the entire structure and admittance of the incident medium (i.e., air), which is represented by (1, 0)^11,13,55^. As shown in Fig. 5a, the effective admittance of the MDM structure for t = 30 nm is equivalent to (0.99, 0.01), which produces near-zero reflection. Meanwhile, the MDM structure for t = 50 nm traces a different path at the resonance wavelength, as marked by the blue curve in Fig. 5b. The dielectric overlay was construed to help suppress the resonant reflection in favor of perfect absorption, while the corresponding slight wavelength shift was mostly ascribed to variations in the reflection phase shift $\phi_{b}$. By making *Y* resemble the admittance of air (1, 0), the reflectance R can be minimized as intended. The reflection coefficients for both the MDM and MDMD structures are accordingly plotted in Fig. S4 of the Supplementary Information. For the proposed MDMD scheme, a near-zero reflection in the vicinity of r (− 0.01, 0.00) was achieved, which led to perfect absorption. Therefore, the overlay satisfies the condition of destructive interference based on a quarter-wave thickness^55^, which indicates that the AR coating layer can significantly prevent reflection and reinforce absorption, even under the condition of a lossless medium integrated with a thick metal layer.

### Discussions on the wavelength and angle dependence of the proposed device

**Figure 6:**
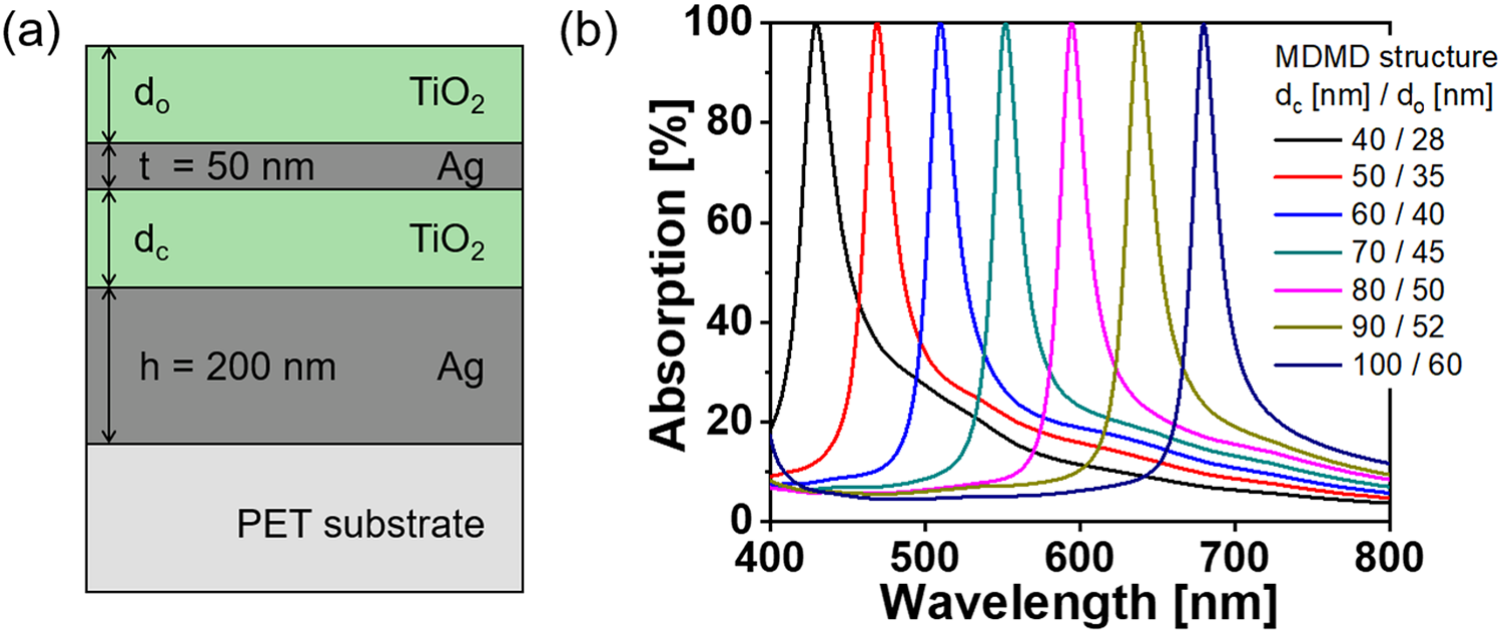
(**a**) Structure of MDMD perfect absorber. (**b**) Calculated absorption spectra for different thin-film perfect absorbers in the visible wavelength region with varying thicknesses of dielectric cavity and overlay.

For practical application of the proposed absorber, we tried various absorbers with varying cavity thicknesses. According to the aforementioned equation, an MDM nano-resonator can flexibly tune the wavelength by tailoring the thickness of the dielectric cavity^1,30^. Figure 6 portrays a suite of device samples with different thicknesses of dielectric layers, where the metal thicknesses (t and h) were fixed. The MDMD perfect absorber is schematically illustrated in Fig. 6a, and the corresponding parameters of the dielectric layers (d_c_ and d_o_) are shown in the legend of Fig. 6b. Regardless of the cavity thickness, near-perfect absorption was preserved for the entire visible band. Table S1 of the Supplementary Information summarizes the parameters for the Ag and TiO_2_ layers, in conjunction with the corresponding resonance wavelengths, FWHMs, and Q-factors. The cavities were designed with thicknesses of 40–100 nm in steps of 10 nm. The thickness of the overlay was chosen assuming an effective admittance of (0, 0). The calculated FWHM varied from 23 to 34 nm, which is equivalent to a Q-factor of 10–30. For comparative analysis, perfect absorbers using an MDM nano-resonator were also considered, as presented in Fig. S5 and Table S2 of the Supplementary Information. They seemed to provide a similar level of absorption as their MDMD-based counterparts, except for a slight discrepancy in the spectral responses. The MDM absorbers realized a maximum Q-factor of 25, which is less than that of the proposed MDMD absorber. Thus, it is revealed that the suggested MDMD scheme achieves a satisfactorily stable perfect absorption in combination with a high Q-factor showing approximately 10–20% enhancement by taking advantage of a dielectric overlay. Higher-order absorption peaks at different wavelengths were observed for thickened dielectric cavities (e.g., d_c_ = 200 nm), as shown in Fig. S6 of the Supplementary Information.

**Figure 7:**
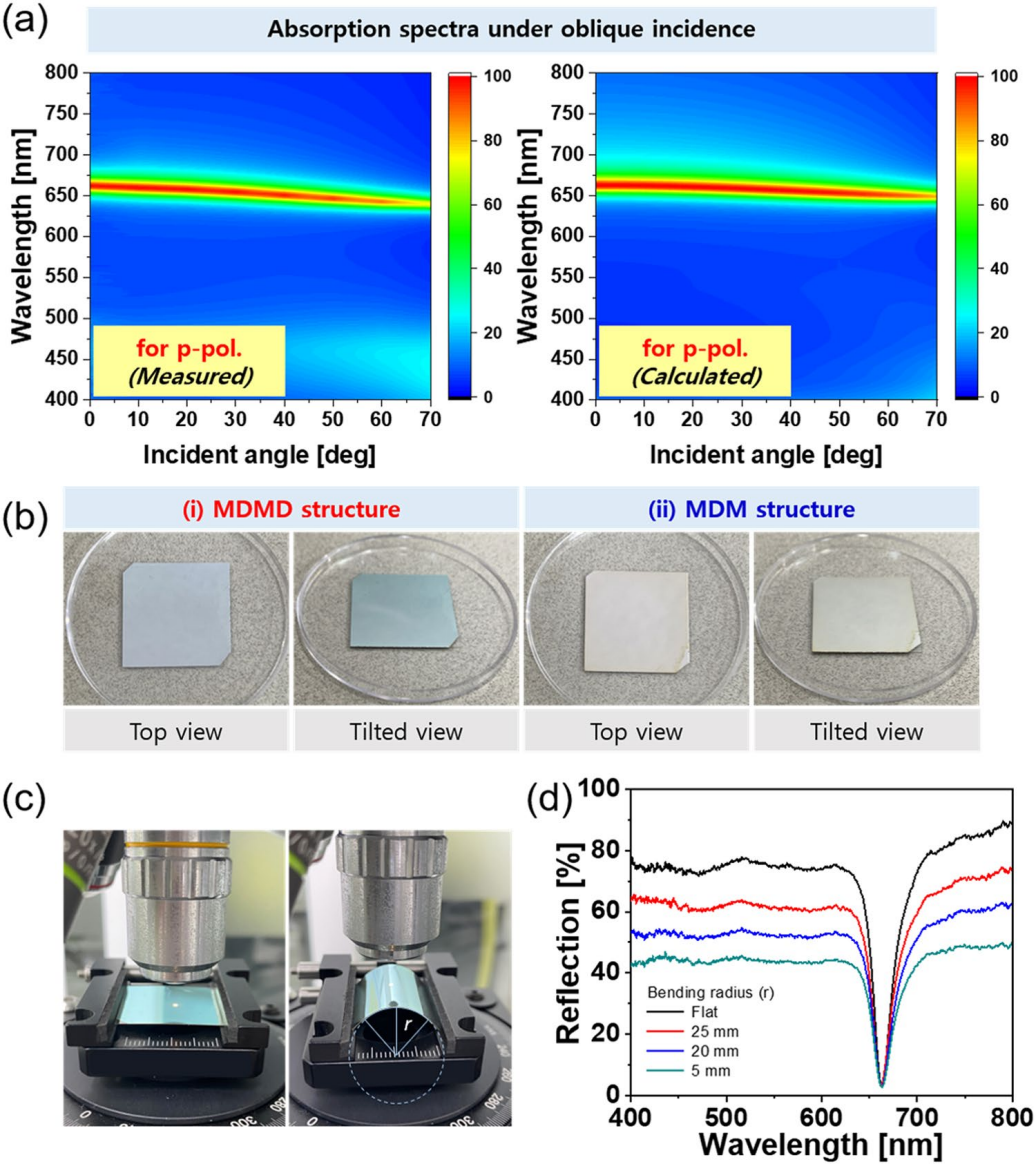
Angular dependence. (**a**) Measured and calculated absorption spectra with oblique incident angles ranging up to 70° for p-polarized light. (**b**) Photographs of the fabricated flexible perfect absorbers incorporating (i) MDMD and (ii) MDM schemes for top and tilted views. (**c**) Experimental set-up for demonstrating consistent absorption for varying curvatures. (**d**) Measured reflection spectra under different bending radii of curvatures.

Finally, we investigated the dependence of the proposed perfect absorber on the angle of incidence under the assumption that such a flexible device will naturally operate under obliquely incident visible light. Thus, a wide angle of acceptance is unequivocally one of the most crucial features. The impact of the incident angle on the optical response was examined. The measured and calculated spectra for p-polarized light are plotted in Fig. 7a; the angle was varied from 0° to 70°. The absorption spectra for s-polarized and unpolarized light are shown in Fig. S7 of the Supplementary Information and indicated that a thin-film nano-resonator is more robust against angle variations of p-polarized light than s-polarized light. The reflection spectra were measured by scanning the incident angle with a spectrophotometer (PHOTON RT, Essent Optics Ltd.), from which the absorption spectra were extracted. Figure 7b shows photographs of the top and tilted views of the fabricated perfect absorber based on the (i) MDMD and (ii) MDM structures. No noticeable color changes were witnessed under oblique incidence. Further, we attempted to explore the performance of the flexible perfect absorber by checking the spectral response depending on the bending radius of curvature. For the absorber mounted on a stage, the reflection spectra were observed by varying the bending radius (r), as shown in Fig. 7c. The results are presented in Fig. 7d; the reflection dips for the resonant wavelength are concretely preserved alongside the corresponding bandwidths, as desired. This suggests that perfect absorption is stably maintained to feature a robust Q factor regardless of the bending of the device. The reflection in the non-resonant spectral band was observed to predictably decline for smaller bending radii, considering that the portion of reflected light, which deviated away from the path of incident light under the bending, could not be captured during the measurement. Nonetheless, the bandwidths for the corresponding measured reflection spectra were kept almost constant. Consequently, the proposed flexible perfect absorber was confirmed to provide a decent angle-insensitive spectral performance irrespective of the bending. Narrowband perfect absorbers are perceived to facilitate applications in diverse fields including imaging, sensors, spectroscopy, and absorption filtering in the visible band^25–27^. In light of its salient benefits like lithography-free production, highly scalable thin-film design, and angle invariant operation, the proposed perfect absorber will be able to categorically catalyze the development of eminent applications such as smart camouflage and stealth^56,57^.

## Conclusion

A perfect absorber is one of the most tangible applications of thin-film nano-resonators. We proposed a flexible perfect absorber based on an MDMD configuration and demonstrated it on a PET substrate. Previously, most thin-film perfect absorbers exploited an MDM scheme by virtue of the Fabry–Pérot resonance, featuring a simple design and high efficiency in the visible band. However, fixing the metal thickness (e.g., 30 nm thick Ag) inhibits the improvement of the Q-factor for such nano-resonators. We developed an MDMD structure by integrating a dielectric overlay atop an MDM nano-resonator. A highly efficient perfect absorber was successfully constructed to deliver an enhanced Q-factor of ~ 33, represented by a narrow bandwidth of FWHM =  ~ 20 nm. The relatively thick Ag layer was effective in attaining a higher Q-factor and smoother surface, while the dielectric overlay facilitated the protection of the Ag layer and a relaxed tolerance during fabrication. The function of the overlay serving as an AR coating, leading to near-zero reflection corresponding to perfect absorption, was meticulously scrutinized according to the optical admittance. For the prospect of their appealing applications, different perfect absorbers with varying thicknesses were also explored. An angle invariant property, which is obviously crucial for such a flexible optical device, was experimentally verified. It is anticipated that the proposed flexible perfect absorber can be widely exploited for implementing advanced sensors and absorption filtering devices geared for smart camouflage and stealth.

## Supplementary information

Supplementary Information.
