# Supplementary material for: Narrowband and flexible perfect absorber based on a thin-film nano-resonator incorporating a dielectric overlay
Source: Sci Rep. 2020 Oct 20;10:17727. doi: 10.1038/s41598-020-74893-1 (PMC7576172; doi:10.1038/s41598-020-74893-1)
Supplement: Supplementary file 1 — Supplementary Information. [file 41598_2020_74893_MOESM1_ESM.pdf]

Supplementary Information

Narrowband and Flexible Perfect Absorber Based on a Thin-film Nano-resonator Incorporating a Dielectric Overlay

Chul-Soon Park[1] and Sang-Shin Lee[1,2,*]

[1]Nano Device Application Center, Kwangwoon University, 20 Kwangwoon-ro, Nowon-gu, Seoul 01897, South Korea

[2]Department of Electronic Engineering, Kwangwoon University, 20 Kwangwoon-ro, Nowon-gu, Seoul 01897, South Korea

[*]Prof. Sang-Shin Lee (slee@kw.ac.kr)

S1: Dispersion characteristics of the materials used for the simulations

Figure S1 shows the dispersion characteristics of the materials (i.e., silver (Ag) and titania (TiO_2)) used to design and analyze the proposed perfect absorber. The refractive indices (n, k) were extracted from deposited Ag and TiO_2 films with an ellipsometer (M-2000D, J. A. Woollam).

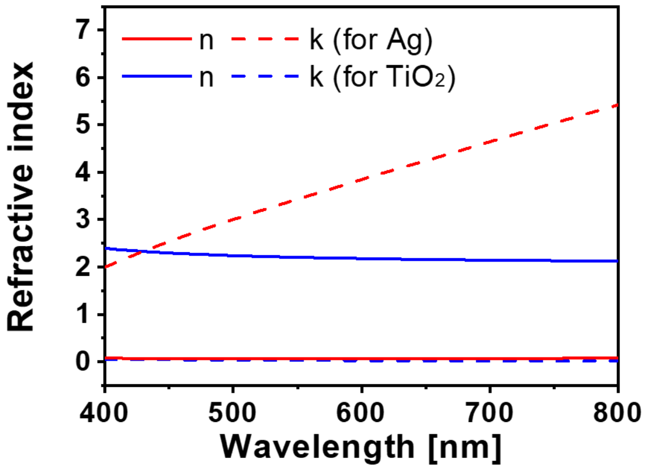

Figure S1. Refractive indices of Ag and TiO_2 films prepared via electron beam evaporation.

S2: Weak absorption relating to a thin-film layer

A thin-film structure comprising a dielectric layer stacked on a sufficiently thick Ag film can be construed as an asymmetric Fabry–Pérot resonator. A spectral resonance can be induced in the dielectric cavity, which entails a certain amount of phase shift at the dielectric–metal interface. As depicted in **Figure S2**, the overall absorption is relatively tenuous for a thin-film structure exploiting a lossless dielectric medium as the cavity because the air–dielectric boundary is supposed to incur no significant reflection.

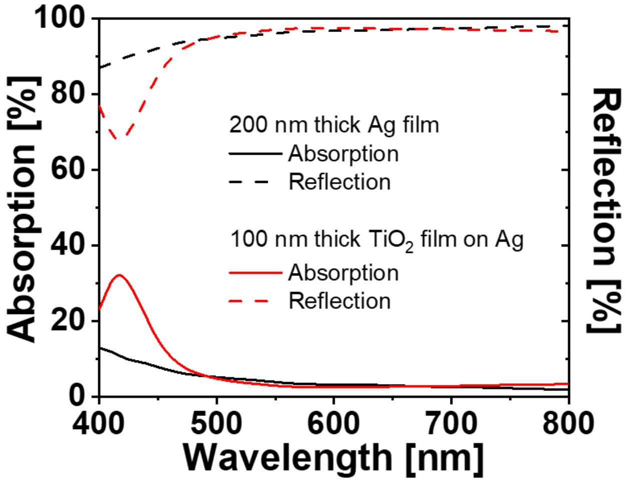

Figure S2. Calculated absorption and reflection spectra for (black curves) 200 nm thick Ag film and (red curves) 100 nm thick TiO_2 film deposited on an Ag substrate.

S3: Absorption spectra for an MDM structure

For the fabricated perfect absorber based on an Ag–TiO_2–Ag nano-resonator, the measured absorption spectra are shown in **Figure S3(a)**; the inset displays the fabricated device. The fabricated device was observed to deliver a slightly broader bandwidth than expected, which may be attributed to a scattering loss originating from the unexpectedly rough surface of Ag, as shown in **Figure S3(b)**.

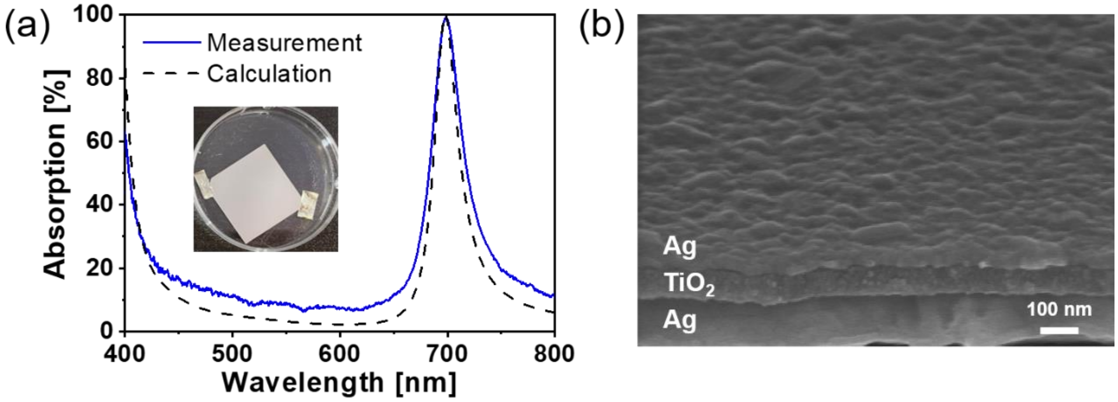

Figure S3. (a) Measured and calculated absorption spectra for an Ag–TiO_2–Ag nano-resonator in the visible wavelength region. (b) Tilted SEM image of the fabricated MDM perfect absorber.

S4: Reflection coefficients for the MDM and MDMD structures

The calculated reflection coefficients according to the optical admittance (Figure 5 in the main manuscript) for the MDM and MDMD structures are plotted in **Figure S4.** The reflection coefficients for both the MDM and MDMD structures ultimately converged at the origin (0, 0), which led to a near-zero reflection equivalent to near-perfect absorption.

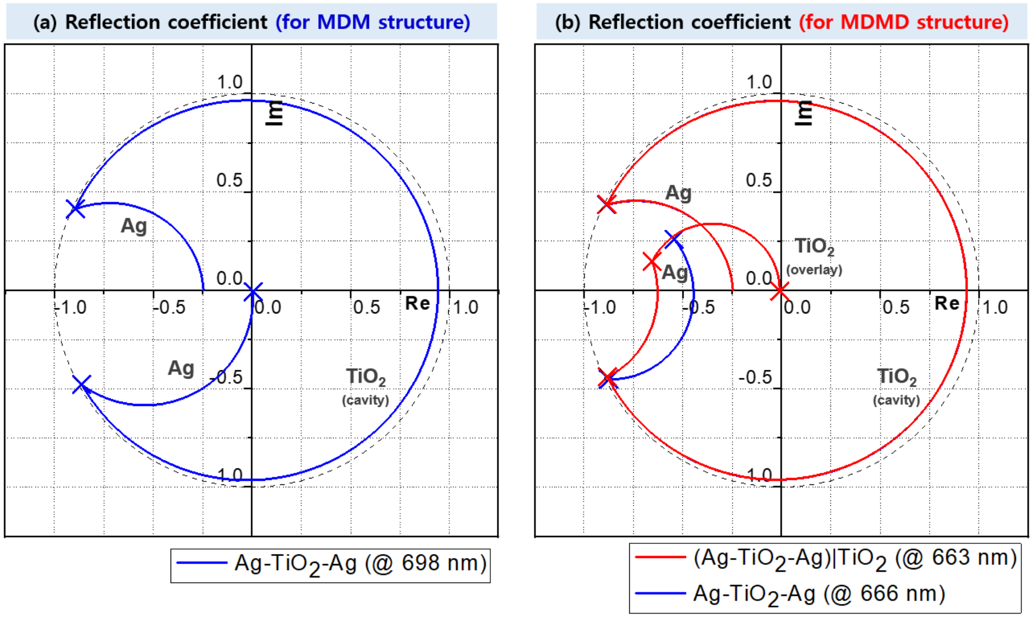

Figure S4. Reflection coefficient diagrams representing the calculated effective admittance for (a) MDM and (b) MDMD structures.

S5: Implementation of different perfect absorbers working at different wavelengths

For an MDM nano-resonator, the resonant wavelength hinges on the thickness of the dielectric cavity. Various perfect absorbers can be created depending on the thickness of the dielectric layers for a fixed metal thickness. For the MDMD structure (Figure 6 in the main manuscript), **Table S1** summarizes the structural parameters in conjunction with performance specifications including the resonance wavelength, full width at half maximum (FWHM), and corresponding Q-factor.

Table S1 | Different perfect absorbers employing an MDMD nano-resonator.

Thickness	Layer	Dev 1	Dev 2	Dev 3	Dev 4	Dev 5	Dev 6	Dev 7
d_o [nm]	TiO_2	28	35	40	45	50	52	60
t [nm]	Ag	50	50	50	50	50	50	50
d_c [nm]	TiO_2	40	50	60	70	80	90	100
h [nm]	Ag	200	200	200	200	200	200	200
Resonance wavelength [nm]		430	469	510	552	595	638	680
FWHM [nm]		34	26	25	23	24	25	23
Q-factor		12.6	18.0	20.4	24.0	24.8	25.5	29.6

The calculated absorption spectra for different thin-film perfect absorbers exploiting an MDM configuration are plotted in **Figure S5**. The detailed parameters, resonant wavelengths, FWHMs, and corresponding Q-factors for MDM nano-resonators are summarized in **Table S2**. For equivalent comparison, the thicknesses of the dielectric cavity (d_c) were set from 40 nm to 100 nm in increments of 10 nm in accordance with the case of the MDMD configuration.

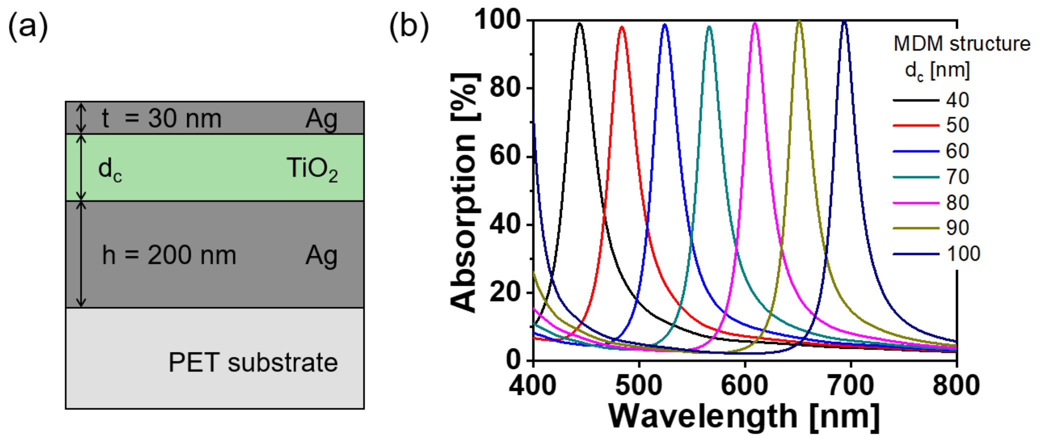

Figure S5. (a) Structure of MDM perfect absorber. (b) Calculated absorption response for different perfect absorbers assuming an MDM configuration for varying thicknesses of dielectric cavity.

Table S2 | MDM nano-resonator based perfect absorbers with a cavity thickness d_c equivalent to the case of the MDMD structure.

Thickness	Layer	Dev 1	Dev 2	Dev 3	Dev 4	Dev 5	Dev 6	Dev 7
**t [nm]**	Ag	30	30	30	30	30	30	30
**d_c [nm]**	TiO_2	40	50	60	70	80	90	100
**h [nm]**	Ag	200	200	200	200	200	200	200
**Resonance wavelength [nm]**		444	484	525	567	609	651	694
**FWHM [nm]**		36	34	32	30	30	28	28
**Q-factor**		12.3	14.2	16.3	18.9	20.3	23.3	24.8

S6: Higher order absorption peaks at different wavelengths

Figure S6 shows the calculated absorption spectra for both MDM and MDMD configuration for a dielectric cavity with d_c = 200 nm. Higher-order absorption peaks for the MDMD structure were observed at different wavelengths with increasing cavity thickness, similar to a typical MDM Fabry-Pérot resonator. Because the TiO_2 overlay was used as an anti-reflection coating for a particular wavelength, it was necessary to properly adjust its thickness to attain near-perfect absorption at the designated wavelength.

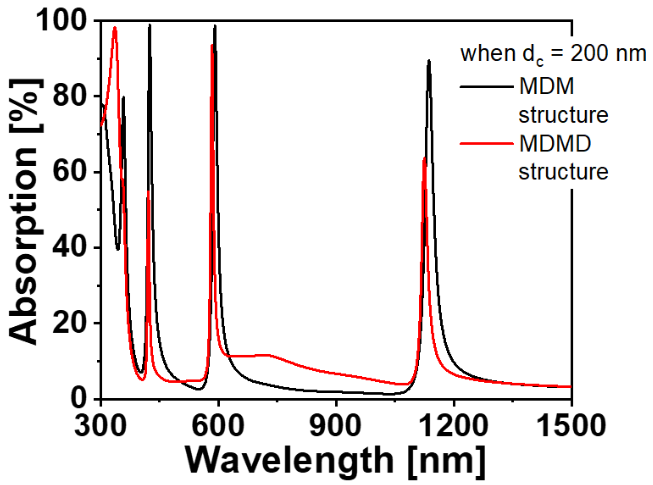

Figure S6. Calculated absorption response for MDM and MDMD configuration for dielectric cavity with d_c = 200 nm.

S7: Polarization-dependent angular tolerance for the proposed absorber

The measured and calculated absorption spectra under oblique incidence are depicted in **Figure S7**. Unlike the spectra under p-polarized light, s-polarized light caused the resonance wavelength to shift in accordance with the incident angle.

The tilted optical admittance, which indicates an optical admittance for oblique incidence, can be denoted as $\eta_p = Y\, cos\, \theta_o \,/\, cos\, \theta$ and $\eta_s = Y\, cos\, \theta \,/\, cos\, \theta_o$ for p- and s-polarization, respectively, where Y is the optical admittance, θ_o is the incident angle from the air, and θ is the propagation angle inside the film [S1, S2]. It is inferred that the tiled admittance for s-polarization is more drastically dependent on the incident angle because of larger variation of $1/\cos\theta_o$than $\cos\theta_o$. Hence, the absorption characteristics of the proposed device, which are directly governed by the optical admittance, are unequivocally presumed to be more sensitive to the incident angle under s-polarization than p-polarization. The angular sensitivity is expected to further reduce by capitalizing on higher-index dielectric cavities [S3].

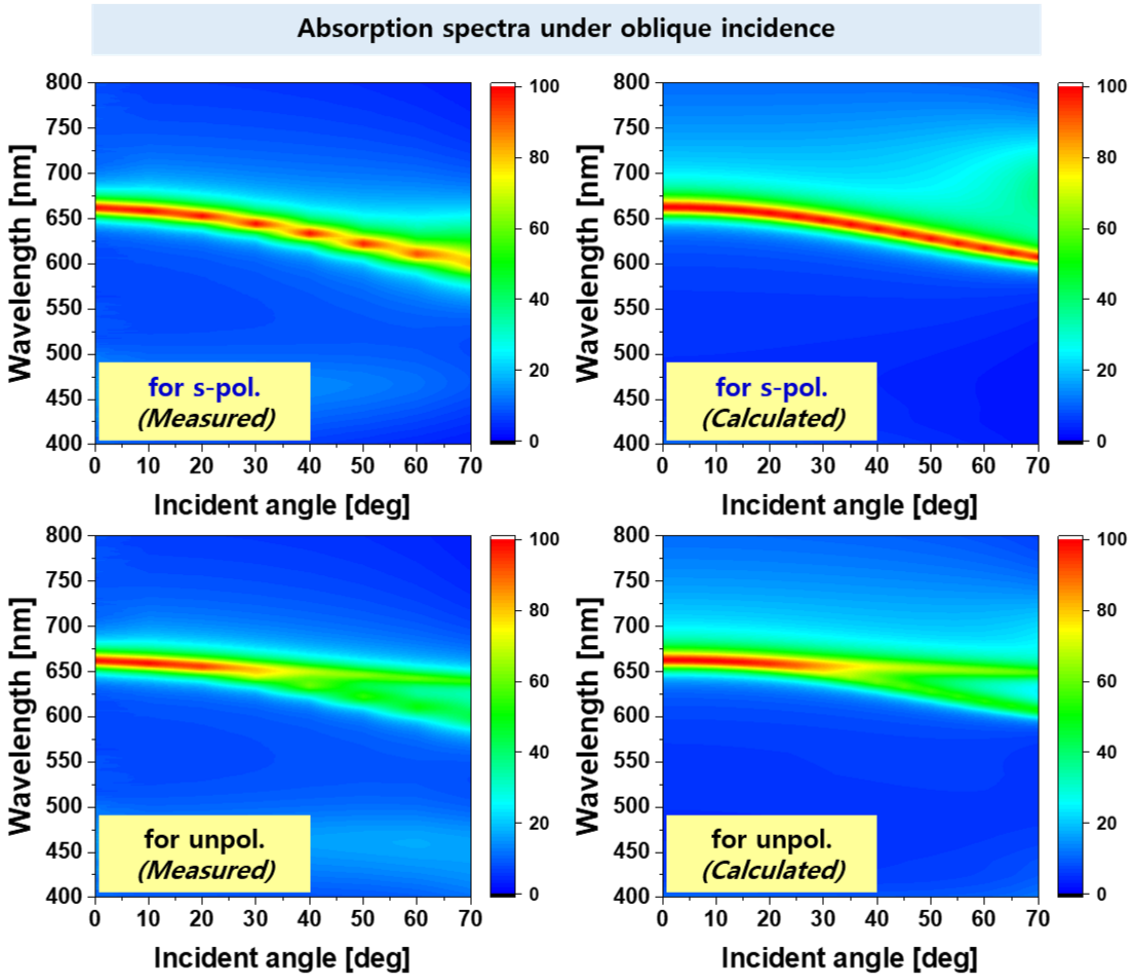

Figure S7. Measured and calculated absorption spectra for oblique incident angles ranging up to 70° for s-polarized and unpolarized light.

References

S1. Macleod, H. A. *Thin-Film Optical Filters*, 4th ed. (CRC Press, 2010)

S2. Park, C. S., Shrestha, V. R., Lee, S. S., Kim, E. S. & Choi, D. Y. Omnidirectional color filters capitalizing on a nano-resonator of Ag-TiO_2-Ag integrated with a phase compensating dielectric overlay. *Sci. Rep.* **5,** 8467 (2015).

S3. ElKabbash, M. *et al.* Iridescence-free and narrowband perfect light absorption in critically coupled metal high-index dielectric cavities. *Opt. Lett.* **42,** 3598−3601 (2017).
